# Purification and Characterization of Endogenous α-Amylase from Glutinous Rice Flour

**DOI:** 10.3390/foods14101679

**Published:** 2025-05-09

**Authors:** Huang Zhang, Fengjiao Zhang, Fengfeng Wu, Lichun Guo, Xueming Xu

**Affiliations:** 1 College of Food and Biological Engineering, Henan University of Animal Husbandry and Economy, No. 6, Longzihu North Road, Zhengzhou 450046, China; 2 The State Key Laboratory of Food Science and Technology, School of Food Science and Technology, Synergetic Innovation Center of Food Safety and Nutrition, Jiangnan University, Wuxi 214122, China

**Keywords:** glutinous rice flour, endogenous α-amylase, purification, characterization

## Abstract

Endogenous α-amylase activity is crucial for determining the end-use value of glutinous rice flour (GRF), and controlling it is a key goal in the milling process. Although the structure and properties of starch and protein in GRF have been extensively studied, there is little information on endogenous α-amylase in GRF. In this study, endogenous α-amylase isolated from GRF was purified and characterized. It was found to have a molecular weight of about 32 kDa, with the highest specific activity at 60 °C and a pH of 6.0. The enzyme is stable below 50 °C and in the pH range of 4.0–7.0. Its activity is Ca^2^⁺-independent but inhibited by Cu^2^⁺, Zn^2^⁺, Mg^2^⁺, Mn^2^⁺, and Ba^2^⁺. Its activity is also reduced by β-mercaptoethanol. The enzyme hydrolyzes amylopectin most efficiently. Circular dichroism spectroscopy showed that the enzyme contains 7.9% α-helix, 35.4% β-folding, 21.1% β-turning, and 35.9% random coils, with a T_m_ value of 63.68 °C. These results suggest that temperature control may be the best strategy for reducing amylase activity in dry-milled GRF, providing a new approach for the development of GRF dry-milling techniques.

## 1. Introduction

α-amylase is a key enzyme in the growth and maturation process of rice and plays a central role in carbohydrate metabolism [1,2,3]. Studies have also demonstrated that α-amylase is initially expressed in the epidermis of cereal seeds and later expressed at high levels in the aleurone layers, and subsequently both tissues can secrete α-amylase into the starchy endosperm and eventually remain in the mature seed [4,5]. This α-amylase is present in all aspects of seed storage, transportation, and processing [6,7,8]. However, high levels of endogenous α-amylase in grains (such as wheat and glutinous rice) can lead to deterioration in the quality of the final product [9,10].

Various α-amylases have been purified and characterized from microbial sources, animal, plants, and their properties. Kumaravel et al. isolated *α*- amylase from the Aspergillus *tamarii* MTCC5152 and found that the enzyme had its optimal activity at a pH of 6.5 and exhibited maximal activity at 55 °C [11]. In order to design specific endogenous α-amylase inhibitors to achieve insect resistance, Thakur purified and characterized α-amylase from Acanthoscelides obtectus (Say) and found that its activity is significantly inhibited by tannic acid, oxalic acid, and HgCl_2_, while Na^+^, Mg^2+^, and Ca^2+^ acted as activators [12]. Singh et al. isolated and purified α-amylase from broad beans and found that its maximal activity was at a pH of 6 and 65 °C [13]. Amylases are extensively used in various industrial processes. To expand their application potential under varying operational conditions, researchers have successfully isolated amylases from microbial exhibiting cold-adapted (optimal temperature at 30 °C) and thermostable (optimal temperature at 80 °C) activity [14,15]. When native amylases fail to meet functional requirements, protein engineering strategies are further used to enhance their catalytic activity and operational stability, thereby tailoring these biocatalysts for specific industrial applications [16,17].

In our previous studies, we found that the content of endogenous α-amylase explained the difference in pasting and rheological properties between wet-milled GRF and dry-milled glutinous rice flour (GRF) [18]. The higher endogenous α-amylase activity in dry-milled GRF increased the degradation of glutinous rice starch molecules during gelatinization, resulting in reduced gel strength, thus leading to the textural deterioration of the final products. Notably, α-amylase inhibition effectively improved the dry-milled GRF product texture. Therefore, understanding the characteristics of endogenous α-amylase is crucial for regulating enzyme activity and developing enzyme inhibitors. However, there is limited information on the endogenous α-amylase in GRF. Thus, it is necessary to purify and characterize this endogenous α-amylase from GRF to develop new strategies to regulate its activity and improve its stability.

This study aimed to extract, purify, and characterize endogenous α-amylase from GRF. The purified enzyme was characterized in terms of its activity at various pH values and temperatures. The molecular weight and secondary structure of the protein were also determined. The findings of this study will pave the way for the development of innovative dry milling techniques to control endogenous α-amylase activity in dry-milled GRF, thereby increasing the possibility of using dry-milled GRF as raw material in food manufacturing.

## 2. Materials and Methods

### 2.1. Materials

All chemical reagents used in this study (3,5 -dinitrosalicylic acid, ammonium sulfate, acetic acid, sodium acetate, disodium hydrogen phosphate, and sodium dihydrogen phosphate; analytically pure) were purchased from Sinopharm Chemical Reagent Co. (Shanghai, China). Unless otherwise indicated, the BCA protein concentration determination kit was obtained from Biyuntian Biotechnology Co. (Shanghai, China).

### 2.2. Purification of Endogenous α-Amylase

The endogenous α-amylase was purified according to the methods of Olajuyigbe with some modifications [19], and the specific operation steps are shown below.

#### 2.2.1. Extraction of Crude Enzyme Solution

The crude enzyme solution was extracted using the following method: Briefly, 100 g of glutinous rice flour was added to 500 mL 0.1 mol/L PBS (a pH of 6.5) buffer and stirred at a low temperature (ice bath, ≤4 °C) for 2 h. The mixture was then centrifuged at 8000 g for 30 min at 4 °C and the supernatant was used for further purification.

#### 2.2.2. Graded Precipitation of Ammonium Sulfate

A certain aliquot of crude enzyme solution was taken, and 10%, 20%, 30%, 40%, 50%, 60%, and 70% (*w*/*v*) of ammonium sulfate was added to the volume of the crude enzyme solution. The mixture was slowly stirred at a low temperature (ice bath, ≤4 °C) for 1 h to prevent the formation of bubbles, then allowed to stand at 4 °C overnight. It was then centrifuged at 10,000× *g* for 30 min at a low temperature. The collected precipitate was resuspended in 0.1 mol/L PBS (pH 6.5) buffer, and the enzyme activity was determined. The concentration with the highest enzyme activity was selected for subsequent experiments. After centrifugation, the precipitate was redissolved in PBS and desalted by dialysis at 4 °C for 12 h, with the dialysate being changed at 1 and 3 h. The dialyzed enzyme solution was then passed through a 0.45 μm aqueous membrane for subsequent purification.

#### 2.2.3. Ion Exchange Chromatography

The DEAE Sepharose Fast Flow ion-exchange chromatography column was pre-equilibrated with 0.1 mol/L PBS (pH 6.5) at an equilibrium flow rate of 1 mL/min. The dialyzed enzyme solution was injected into the AKTA Purifier UPC system (GE Healthcare Life Sciences, Marlborough, MA, USA). After the sample loading was completed, isocratic elution was performed with 0.1 mol/L PBS (a pH of 6.5) containing 0.5 mol/L NaCl. Based on the results of the linear elution, gradient elution was then performed with PBS (0.1 mol/L, a pH of 6.5) containing 0, 0.1, 0.2, 0.3, 0.4, and 0.5 mol/L NaCl at a controlled flow rate of 3 mL/min and a collection rate of 1 min/tube. The absorbance of the eluate was measured at 280 nm using a UV detector. The enzyme activity, volume, and protein content of the liquid in the peak portion of the collected fractions were detected. Fractions containing endogenous α-amylase activity were pooled.

#### 2.2.4. I Gel Filtration Chromatography

The Superdex-200 gel column (GE Healthcare Life Sciences, Marlborough, MA, USA) was pre-equilibrated overnight with 0.1 mol/L PBS (pH 6.5). After ion exchange chromatography, the enzyme solution was injected into the system and eluted with 0.1 mol/L PBS (pH 6.5) buffer at a flow rate of 3.5 mL/min. The amylase activity of each peak was measured, and the active fractions were collected and concentrated by ultrafiltration. These fractions were then subjected to SDS-PAGE, and fractions with the same bands were pooled to determine the volume, enzyme activity, and protein content.

### 2.3. Determination of Protein Content

The BCA kit was used to determine the amount of protein in the enzyme solution.

### 2.4. Determination of Endogenous α-Amylase Activity

Amylase activity was determined using the 3,5-dinitrosalicylic acid (DNS) method [20], with a standard curve plotted using glucose. The substrate solution was prepared with 0.1 mol/L acetate buffer (pH 6.0) and 1% (*w*/*v*) soluble starch, thoroughly mixed. One milliliter of the substrate solution and 0.1 mL of appropriately diluted enzyme solution were reacted at 37 °C for 30 min. The reaction was terminated by adding 1.5 mL of DNS reagent. After 5 min in a boiling water bath, the reaction mixture was cooled by transferring it to ice water. Absorbance values were then determined at 540 nm. The blank control was distilled water. One unit of enzyme activity was defined as the amount of enzyme that releases 1 μmol of glucose from 1% soluble starch per minute.

### 2.5. SDS-PAGE and Enzyme Activity Staining Analysis

The molecular weights of purified enzymes were analyzed by SDS-PAGE. Enzyme solutions from each purification stage were analyzed using a 5% separating gel and a 12% stacking gel [21]. Then, 40 µL of enzyme solution was mixed with 10 µL of loading buffer (Tris-HCl, 0.125 mol/L, pH 6.8, containing 2% SDS (*w*/*v*), 0.05% (*w*/*v*) bromophenol blue, and 10% glycerol), homogenized, and then loaded onto the gel. The electrophoresis buffer was cooled to 4 °C in a refrigerator before loading. Electrophoresis was carried out at 4 °C, and the run was stopped after 80 min at a constant voltage of 120 V. The gel was removed, washed twice with deionized water, and then stained with Coomassie Brilliant Blue. The sample contained 5% 2-*Me* under reduction conditions.

After electrophoresis, the gel was washed twice with deionized water and immersed in 0.1 mol/L PBS buffer (a pH of 6.8) containing 0.5% (*v*/*v*) Triton X-100 for 30 min to remove SDS. The gel was then overlaid with a starch agar plate containing 1% starch and incubated at 37 °C for 2 h. The gel was stained with 0.1% I2/1% KI solution. The transparent areas on the brown background were identified as regions with endogenous α-amylase activity.

### 2.6. Optimal pH for Endogenous α-Amylase

A 1% soluble starch solution was prepared using buffers of 0.1 mol/L acetate (a pH of 3.0–6.0), phosphate (a pH of 7.0–8.0), Tris-glycine (a pH of 9.0–10.0), and carbonate (a pH of 10.0–11.0). The enzyme activity was determined under different pH conditions using the method described in Section 2.4. The relative activity of endogenous α-amylase at different pH values was calculated using the highest enzyme activity as 100%.

### 2.7. pH Stability of Endogenous α-Amylase

The stability of endogenous α-amylase was measured using the following method. Buffers of 0.1 mol/L acetate (a pH of 4.0–6.0), phosphate (a pH of 7.0–8.0), and Tris-glycine (a pH of 9.0) were prepared, each containing a 1% soluble starch solution. The endogenous α-amylase was incubated in a water bath at 37 °C for 120 min under different pH conditions. The residual activities of endogenous α-amylase at 30 min and 120 min were determined using the method described in Section 2.4 and compared with those of their respective controls incubated for 0 min (100%). The stability of the endogenous α-amylase was calculated at each pH.

### 2.8. Optimal Temperature for Endogenous α-Amylase

The relative enzyme activity of endogenous α-amylase at different temperatures was calculated using the method in Section 2.4, where the enzyme activity of endogenous α-amylase was determined at 30 °C–90 °C and the highest enzyme activity was taken as 100%.

### 2.9. Temperature Stability of Endogenous α-Amylase

The substrate and enzyme solution were incubated at 30 °C, 40 °C, 50 °C, 60 °C, and 70 °C for 120 min. The thermal stability of endogenous α-amylase in the range of 30–70 °C was determined using the method described in Section 2.4 to measure the residual enzyme activity at 30 min and 120 min. The enzyme activity at 0 min of their respective incubations was used as a control (100%).

### 2.10. The Effect of Metal Ions on the Activity of Endogenous α-Amylase

The effect of metal ions on the activity of endogenous α-amylase was determined using the following method: Cu^2^⁺, Ca^2^⁺, Zn^2^⁺, Mg^2^⁺, Mn^2^⁺, and Ba^2^⁺ were added to the reaction system to achieve concentrations of 1 mmol/L and 5 mmol/L [22]. The method described in Section 2.4 was used to determine the effect of these different metal ions on the activity of endogenous α-amylase. The control was the enzyme activity (100%) in the absence of metal ions.

### 2.11. The Impact of Various Compounds on the Activity of Endogenous α-Amylase

DTT, EDTA, and 2-Me were added to the reaction system to achieve concentrations of 1 mmol/L and 5 mmol/L. SDS, Tween 20, and Triton X-100 were added to achieve concentrations of 0.1% and 0.5% (*w*/*w*) in the system [23]. The effects of these different compounds on the activity of endogenous α-amylase were determined using the method described in Section 2.4. The enzyme activity in the absence of these compounds was used as the control (100%).

### 2.12. Substrate Specificity of Endogenous α-Amylase

The relative enzyme activities of endogenous α-amylase under different substrates were determined using the method described in Section 2.4. The substrates used were 0.5% soluble starch, maize starch, tapioca starch, wheat starch, potato starch, and branched-chain starch. Soluble starch was used as the control (100%).

### 2.13. Circular Dichroism Spectroscopy Determination

Endogenous α-amylase was configured with 20 mmol/LPBS buffer (a pH of 6.5) to a concentration of 0.2 mg/mL in the sample. Far-UV CD spectra were recorded from 260 nm to 190 nm at 25 °C using a 1 mm cuvette. For secondary structure unwinding temperature analysis, protein samples were heated from 25 °C to 95 °C, ramping up at a heating rate of 0.5 °C/min to record CD spectra at 220 nm [21]. The obtained data were calculated by CDDD 2.1 (Applied Photophysics Ltd., Leatherhead, UK) and Chirascan Global3 (Applied Photophysics Ltd.) software. Pyrolytic folding was fitted using a sigmoid curve.

## 3. Results and Discussion

### 3.1. Isolation and Purification of Endogenous α-Amylases

Amylase is usually present in soluble form in cereals, which facilitates its separation from the grain. Amylase is usually extracted from grains using buffers (e.g., phosphate, acetate, and Tri-HCl), and the extracted crude enzyme solution is then concentrated by graded precipitation using ammonium sulfate or organic solvents (ethanol or acetone). This step is carried out at low temperatures (4 °C) to minimize the loss of enzyme activity during extraction.

Ion-exchange chromatography is a technique used for the separation of proteins based on their net charge and has been applied to the separation of amylases from most cereals. The enzyme fraction obtained by ion-exchange chromatography is further separated, based on size, by molecular sieve chromatography to obtain a purified enzyme solution. In this study, amylase was separated and purified from GRF using low-temperature extraction with phosphate-buffered saline (PBS, a pH of 6.5) solution, ammonium sulfate precipitation, ion-exchange chromatography, and molecular sieve chromatography. The enzyme activity levels, enzyme recovery rates, and multiplicities in each purification step are shown in Table 1. During the purification process, the purification multiplicity of purified endogenous α-amylase isoforms was increased by 2.72-fold after ammonium sulfate precipitation and the multiplicity of the enzyme was increased by 6.11-fold after anion-exchange chromatography, relative to the crude enzyme fraction. After purification by Superdex-200, the recovery rate was 8.78% and the multiplicity of the enzyme was 33.0-fold.

### 3.2. Molecular Weight Determination

**Figure 1:**
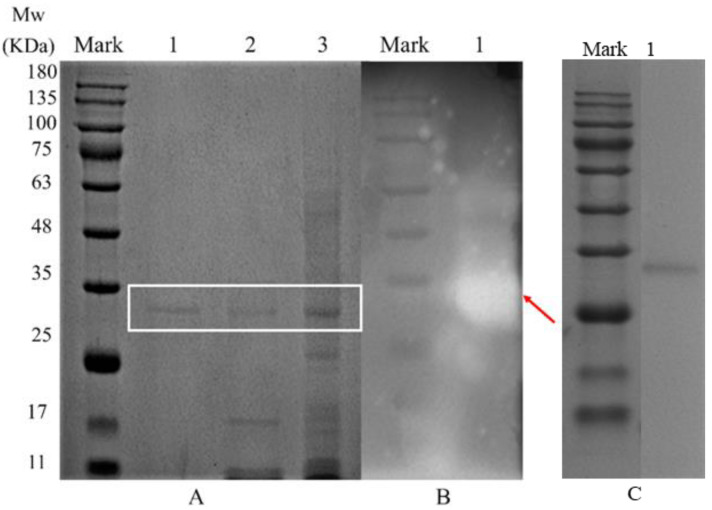
(**A**) SDS-PAGE of purified α-amylase under non-reducing condition. Lane 1: Superdex-200 eluate; lane 2: DEAE Sepharose Fast Flow eluate; lane 3: ammonium sulfate precipitation; (**B**) Zymogram showing α-amylase activity. Line 1: purified α-amylase; (**C**) Superdex-200 SDS-PAGE under reducing conditions.

After each purification step, electrophoresis was performed under reducing and non-reducing conditions in order to determine the molecular size, native structure and activity of endogenous α-amylase (Figure 1). Electrophoretic protein separation analysis after ammonium sulfate precipitation, DEAE Sepharose Fast Flow ion-exchange column chromatography and Superdex-200 size exclusion column chromatography purification revealed a single protein band (Figure 1A), indicating that a purer endogenous α-amylase had been obtained. Furthermore, on sodium dodecyl sulfate-polyacrylamide gel electrophoresis (SDS–PAGE) under reducing conditions, the purified α-amylase gave a single protein band corresponding to a molecular mass of approximately 32 kDa (Figure 1C). After electrophoresis, the gel was spread flat on an agar plate containing starch and incubated at a suitable temperature to verify that the protein band corresponding to endogenous α-amylase had amylase activity. Native-PAGE profile, using the starch–iodine method to detect the presence or absence of amylase, showed a protein band corresponding to amylase activity staining (Figure 1B), which appears as a transparent area on the gel (at the red arrow), in which starch was hydrolyzed by amylase, further confirming that the 32 kDa band is endogenous α-amylase. Usually, the reported molecular weight of cereal amylases ranges from about 20 to 84 kDa [24,25,26], which is consistent with the molecular weight of the endogenous α-amylase obtained in this study.

### 3.3. Optimum pH of Endogenous α-Amylase

**Figure 2:**
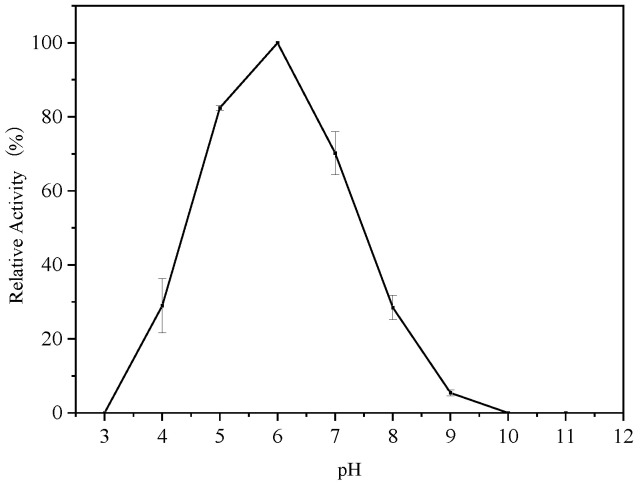
Effect of pH on the endogenous α-amylase activity.

Enzyme activity is strongly influenced by the pH. The optimum pH of endogenous α-amylase from cereals is around 6.0 and may vary slightly from one source to another [26]. The relative activity of endogenous α-amylase in glutinous rice flour at each pH, shown in Figure 2, reveal that the enzyme activity was detected in the pH range of 4.0–9.0, and the endogenous α-amylase had its highest activity at a pH of 6.0 and was stable at a pH of 5.0–7.0, maintaining more than 70% enzyme activity. However, the activity of endogenous α-amylase decreased to about 30% at pH values of 4.0 and 8.0. No enzyme activity was observed with a further decrease (pH of 3.0) or increase (pH of 10.0 and pH 11.0) in pH values, suggesting that the enzyme might have been inactivated. The pH plays a crucial role in the structural stability of the enzyme, and amylase is irreversibly inactivated under extreme pH conditions [27]. Under conditions close to the optimum active pH, small changes in the pH may lead to changes in the group ions in the active site leading to changes in the structure of the enzyme. However, the primary structure of the amylase may not change much, and its activity may be only slightly altered. At a pH far from the isoelectric point of the protein, electrostatic repulsion between similar charges formed by electrons of the functional groups tends to unfold, leading to denaturation and inactivation of the enzyme protein [28].

### 3.4. The pH Stability of Endogenous α-Amylases

**Figure 3:**
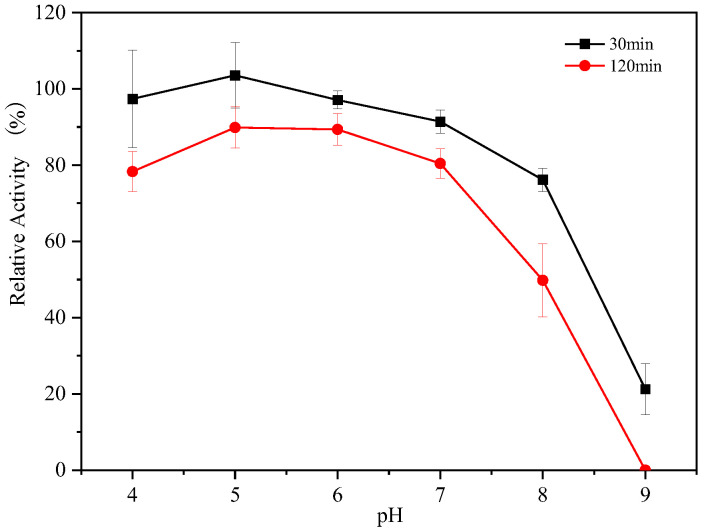
The effect of pH on enzyme activity.

The stability of endogenous α-amylases under different pH conditions was evaluated by incubating the endogenous α-amylases for 120 min in buffers at different pH values, with a non-incubated sample serving as a control. As shown in Figure 3, at 30 min, high enzyme activity was maintained throughout the pH range of 4.0–8.0, and the stability of endogenous α-amylase decreased sharply at a pH of 9.0. After 120 min of incubation, the residual enzyme activity decreased to different degrees compared with that of 30 min, and more than 80% of the residual enzyme activity was observed in the pH range of 5.0–7.0, indicating that the stability of endogenous α-amylase was high in this pH range. The residual enzyme activity decreased significantly after a pH > 7.0, and the enzyme activity could not be detected in the pH at 9.0. This result indicates that endogenous α-amylase is unstable under alkaline conditions and highly stable under acidic conditions.

### 3.5. Optimum Temperature of Endogenous α-Amylase

**Figure 4:**
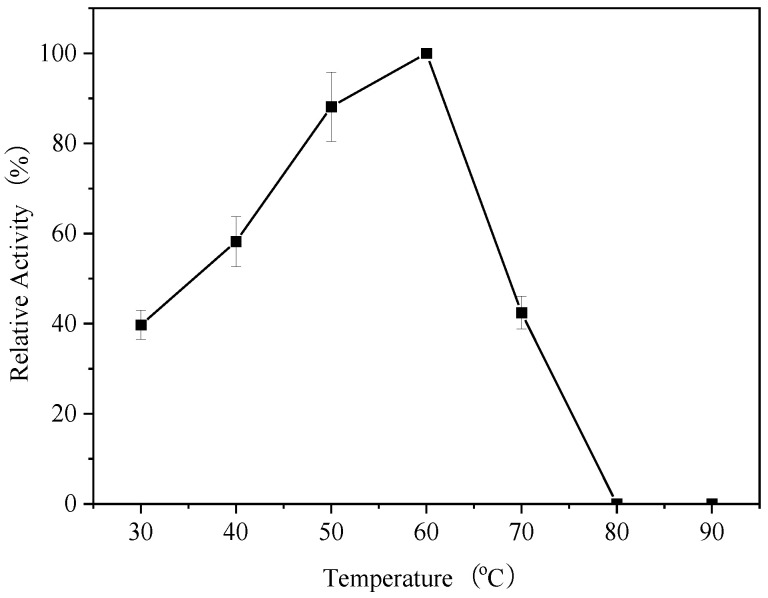
Effect of temperature on the endogenous α-amylase activity.

To determine the effect of temperature on the activity of endogenous α-amylase in glutinous rice, the enzyme activity was determined at temperatures ranging from 30 to 90 °C (Figure 4). At 30 °C, the activity of endogenous α-amylase was around 40%, and gradually increased with increasing temperature, showing its highest activity at 60 °C. Increasing the temperature to 70 °C decreased the activity of endogenous α-amylase to around 40%, and further increasing the temperature led to the complete inhibition of endogenous α-amylase enzyme activity. Within the range of 30 to 60 °C, the reaction rate of the endogenous α-amylase increased with the rise in temperature. This is due to the increase in the kinetic energy of the molecules by the high temperature, enabling more substrate molecules to reach the activation energy threshold. The enzyme activity began to decrease when the temperature exceeded 60 °C, and although the rate of the enzymatic reaction was increased at this time, the structure of the protein was destroyed at high temperature, resulting in a rapid decrease in amylase activity. The enzyme activity decreased rapidly, and no activity was observed when the temperature exceeded 70 °C, at which time the enzyme may have been completely inactivated. Bertoft [27] reported that the optimum temperature for endogenous α-amylase activity in wheat was between 50 and 70 °C, which is consistent with our results.

### 3.6. Thermal Stability of Endogenous α-Amylases

**Figure 5:**
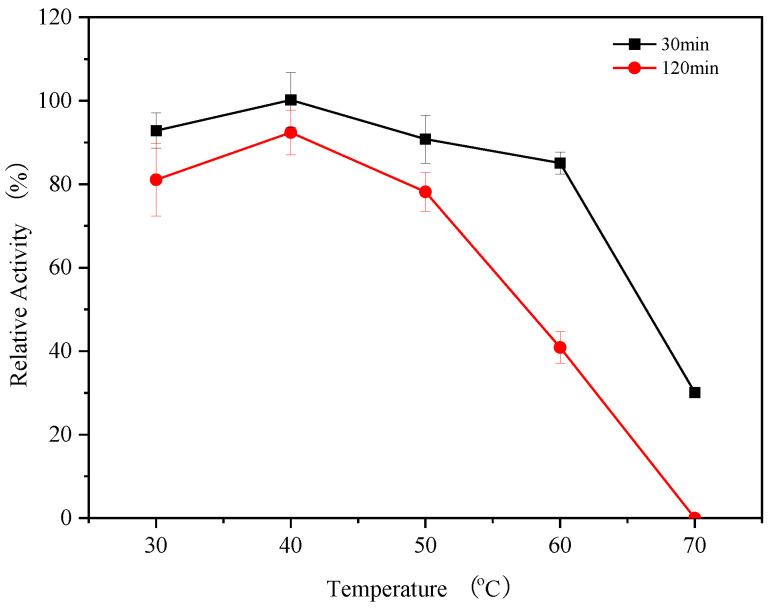
The effect of temperature on enzyme activity at different temperatures.

The results of the thermal stability analysis of endogenous α-amylases, shown in Figure 5, reveal that the residual activity of endogenous α-amylase remained above 80% in the temperature range of 30–60 °C for 30 min, indicating the high stability of endogenous α-amylases in this temperature range. However, at 70 °C, the endogenous α-amylases lost about 70% of their enzyme activity within 30 min. After incubation for 120 min, the residual enzyme activity of all α-amylase isozymes decreased to different degrees compared with their activity at 30 min, but still retained high activity in the range of 30–50 °C. At 60 °C, only 40% of the α-amylase enzyme activity remained, and no enzyme activity was detected at 70 °C. The residual enzyme activity of α-amylases from *Bacillus licheniformis* after 120 min at 60 °C and 70 °C was 86 and 66.7%, respectively [20]. These results showed that prolonged exposure to a temperature of 60 °C can easily lead to the inactivation of endogenous α-amylase exhibiting marked thermal sensitivity.

### 3.7. Effect of Metal Ions on Endogenous α-Amylase Activity

**Figure 6:**
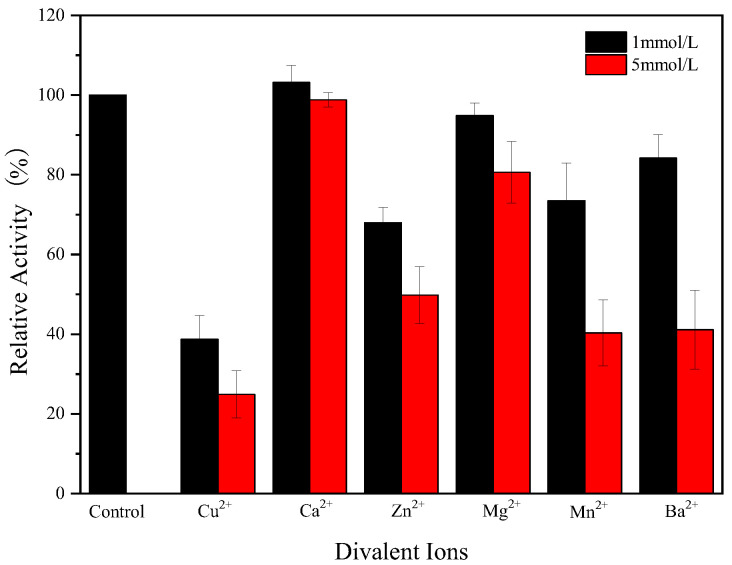
Effect of metal ion on the endogenous α-amylase activity.

Metal ions affect the structure of the enzyme and thus the catalytic activity of amylase. As shown in Figure 6, Ca^2+^ did not have a significant effect on the endogenous α-amylase activity at both concentrations tested, Cu^2+^ significantly inhibited the amylase activity of endogenous α-amylase, whereas Zn^2+^, Mg^2+^, Mn^2+^, and Ba^2+^ also inhibited the amylase activity. Endogenous α-amylase maintained 80% activity in 1 mmol of Mg^2+^, Ba^2+^, and 5 mmol of Mg^2+^, but high concentrations of Ba^2+^ (5 mmol) resulted in a substantial decrease in enzyme activity. In general, α-amylase shows a good affinity for Ca^2+^, and the binding of α-amylase molecules to Ca^2+^ maintains the conformational stability of amylase molecules, as a result, Ca^2+^ usually promotes amylase activity. However, in this study, calcium ions had no significant effect on amylase activity, implying that the endogenous α-amylase in glutinous rice flour is a non-Ca^2+^ dependent amylase. To date, many Ca^2+^-independent amylases have been identified by multiple studies [29,30].

The strong inhibition of amylase activity by Cu^2+^ may indicate that amino acid residues with sulfhydryl, carboxyl, and indole groups may be associated with amylase activity. Mn^2+^ inhibits the catalytic activity of amylase, and a similar phenomenon was found by Sudan [21], which may be related to the structure of amylase. Overall, all metal ions tested except Ca^2+^ inhibited the amylase activity, suggesting poor compatibility between the endogenous α-amylase and metal ions in glutinous rice flour.

### 3.8. Effect of Compounds on Enzyme Activity

**Figure 7:**
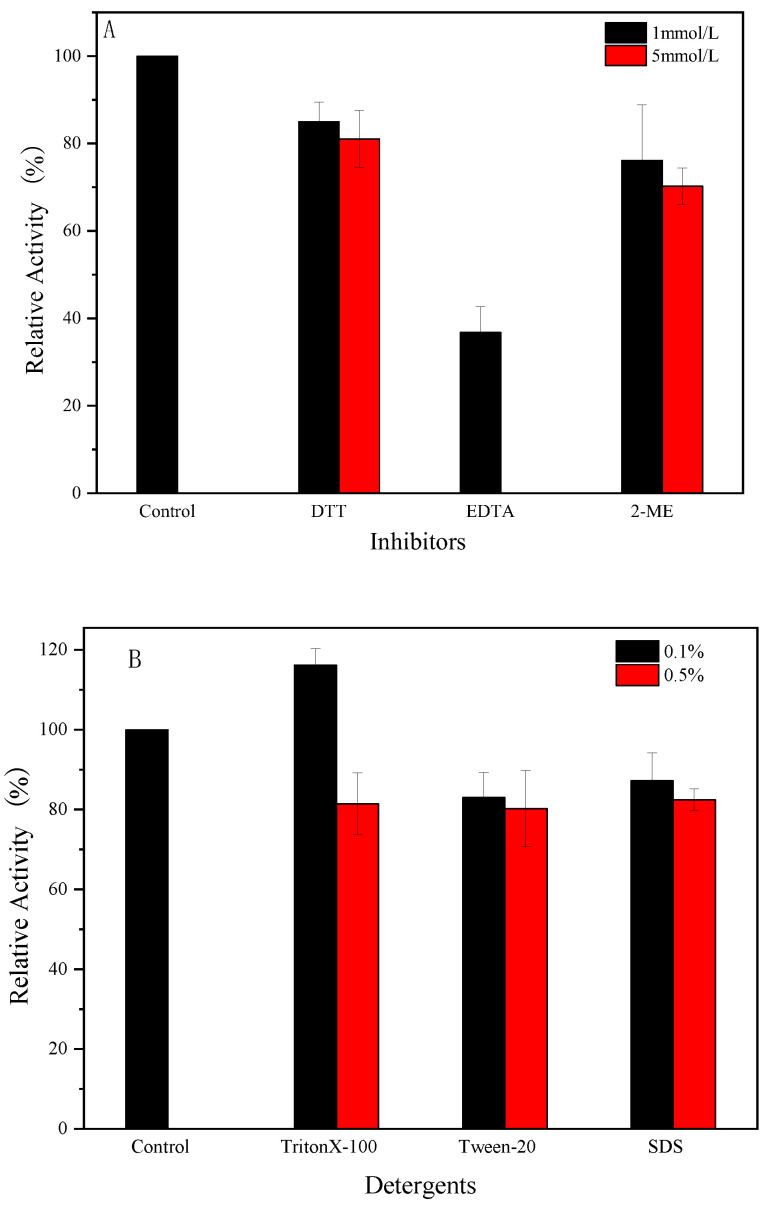
Effect of various modulators on α-amylase activity. (**A**) Inhibitors; (**B**) detergents.

In physicochemical characterization, different chemical compounds are usually used for the pretreatment of glutinous rice flour. The study of the effects of various chemical compounds on the activity of endogenous α-amylase in glutinous rice flour can provide a theoretical reference for the selection of suitable treatment methods for glutinous rice flour. As shown in Figure 7, the chelating agent ethylenediaminetetraacetic acid (EDTA) strongly inhibited the endogenous α-amylase activity, especially at a high concentration (5 mmol), at which no amylase activity was observed. This finding suggests that endogenous α-amylase may be a metalloenzyme. There may be one or more high-affinity metal binding sites in the α-amylase protein molecule that release metal ions in the presence of EDTA, leading to a decrease in the catalytic activity of the enzyme. Among the reducing agents, endogenous α-amylase maintained about 70 and 80% or more of its activity in the presence of 2-mercaptoethanol and dithiothreitol (DTT), respectively. The disulfide bond is an important structure for maintaining the protein conformation. The disruption of disulfide bonds leads to alteration in the protein structure and thus its activity. The inhibition of endogenous α-amylase activity by reducing agents may imply that disulfide bonds are present in endogenous α-amylase and that the active center of the enzyme requires the disulfide bonds to maintain the conformation of its active site. In previous studies, DTT was found to act as a specific inhibitor of cysteine disulfide bond formation, inhibiting the enzyme by breaking intramolecular and intermolecular disulfide linkages [31]. Some amylases can maintain 97% activity in the presence of DTT [32], but the presence of DTT in this study significantly reduced the enzyme activity, indicating that cysteine residues are involved in catalysis and located at the active site of the enzyme.

Surfactants usually alter the charge distribution on the surface of proteins, disrupting their secondary and tertiary structures, thereby altering some of their properties. The non-ionic surfactant TritonX-100 had no significant effect on endogenous α-amylase activity at a concentration of 0.1% and inhibited its catalytic activity at a concentration of 0.5%. The anionic surfactant SDS and non-ionic surfactant Tween-20 both inhibited endogenous α-amylase to varying degrees, and its retained approximately 80% of its enzyme activity at low concentrations of SDS and Tween-20.

### 3.9. Substrate Specificity of Endogenous α-Amylase

**Figure 8:**
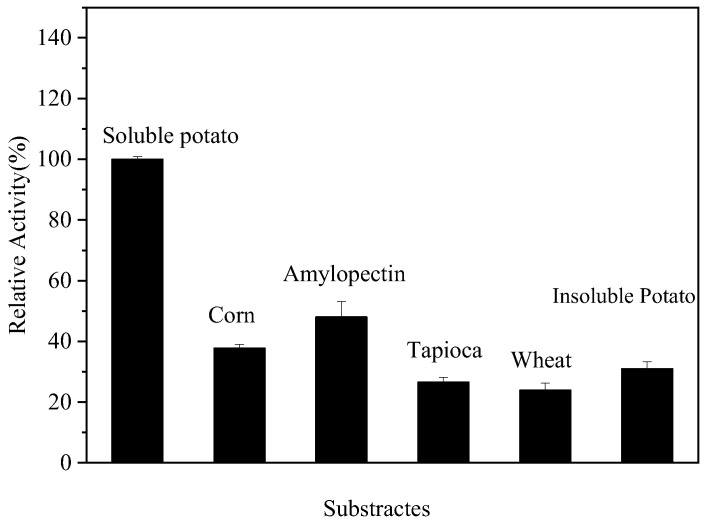
Relative substrate hydrolysis.

The relative rate of hydrolysis of the substrate by α-amylase under different conditions was determined using raw starch from various sources as substrate and the hydrolysis rate of soluble starch as a control. The results, which are shown in Figure 6, Figure 7 and Figure 8, revealed that the relative hydrolysis rates of maize starch, branched-chain starch, tapioca starch, wheat starch, and potato starch with endogenous α-amylase were 37.78, 48.06, 26.57, 23.93, and 30.97%, respectively. Also, the endogenous α-amylase was able to hydrolyze a wide range of substrates by hydrolyzing all the tested substrates. The highest relative hydrolysis rates were observed with branched-chain starches and the lowest with wheat starches. Guillaum et al. [33] reported that Hermetia illucens α-amylase showed maximum activity with rice starch. Also, the rate of hydrolysis of starch was found to be affected by the ratio of branched/straight chain starch molecules in starch, with branched starch molecules being more susceptible to attack by α-amylase in starch granules. In addition, the hydrolysis rate of starch was also found to be affected by the size of starch granules, starch damage, the type of crystal structure of starch, the branch chain length of starch molecules, and the size of starch molecules. Thus, the finding that endogenous α-amylase showed different hydrolysis rates with different substrates may be related to these factors.

### 3.10. Circular Dichroism (CD) Spectroscopy Analysis

**Figure 9:**
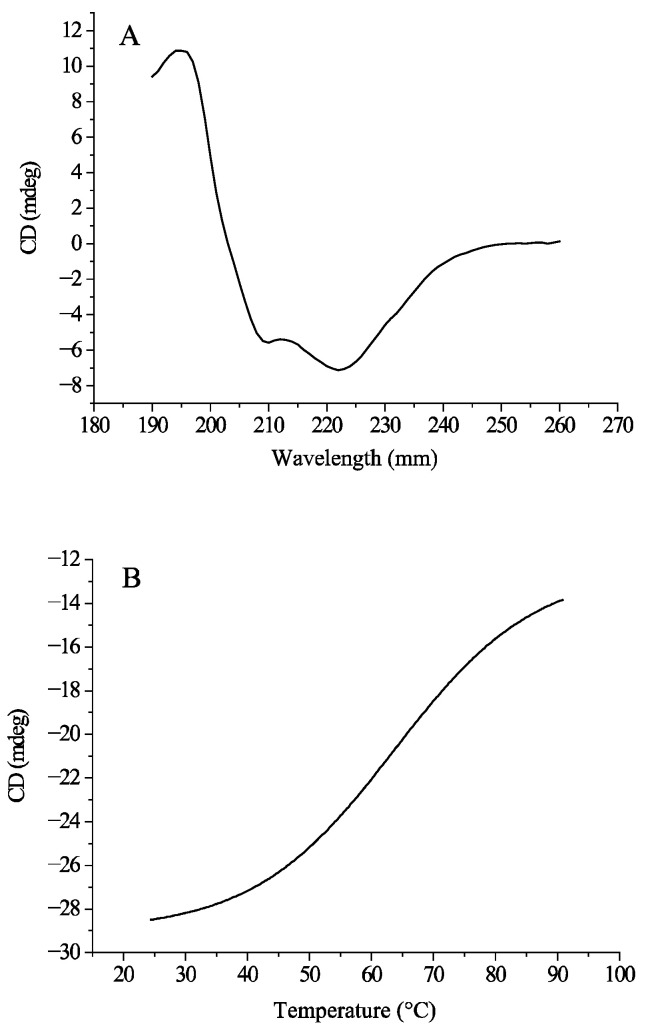
CD spectra of purified endogenous α-amylase. (**A**) CD spectra of endogenous α-amylase was recorded from 260 nm to 190 nm; (**B**) CD spectra of purified endogenous α-amylase at 220 nm at increased temperature from 25 °C to 95 °C.

Different secondary structures have unique circular dichroism (CD) spectra at specific wavelengths, so the secondary structure of amylase and its variations can be studied by CD spectral analysis. Figure 9 show the CD spectra of the endogenous α-amylase solution. In the wavelength-scanned spectrum (A), two peaks appeared near 222 nm and 208 nm, which were due to by the π → π* and n → π* transitions of the amide group, which is typical of α-helical structures. Similar results were previously observed in α-amylase [34]. In addition, the band at 222 nm has a higher intensity than that at 208 nm, suggesting the presence of α/β structural domains in the endogenous α-amylase structure [35]. The relative contents of the four secondary structural motifs of endogenous α-amylase, namely α-helix, β-folding, β-turning, and random coils, were calculated by CDNN 2.1 software to be 7.9, 35.4, 21.1, and 35.9%, respectively.

The effect of temperature on the secondary structure of the proteins was investigated by high-temperature scanning at 220 nm. The enzyme conformation transition temperature is usually expressed as T_m_, with lower Tm values indicating poorer stability [36]. In the fitting curve of the thermal unfolding spectrogram (B), the transition T_m_ of endogenous α-amylase was determined to be 63.68 °C, and the T_m_ revealed that the secondary structure of the endogenous enzyme begins to thermally unfold at 63.68 °C, and the secondary structure irreversibly changes, which ultimately leads to the inactivation of the enzyme. Therefore, the activity of endogenous α-amylase may be greatly reduced at 63.68 °C and above [37,38].

## 4. Conclusions

In this study, α-amylase was isolated and purified from GRF and characterized. Endogenous α-amylase was found to have a molecular mass of about 32 kDa. The optimum temperature and pH for the amylase was 60 °C and a pH of 6, respectively. The enzyme was also found to have high activity over a wide range of pH values (4.0–7.0). Notably, endogenous α-amylase has been demonstrated to be labile at a high temperature, and its secondary structure conformation changes when the temperature exceeds 63.68 °C. The findings of this study highlight that heat treatment may have the potential to inhibit enzyme activity. This study will be extended to investigate the heat treatment of GRF at different temperatures and evaluate its inhibitory effect on α-amylase activity.

## Figures and Tables

**Table 1:** Purification of α-amylase from glutinous rice flour.

**Procedure**	**Total** **Activity/U**	**Total** **Protein/mg**	**Specific** **Activity U/mg**	**Recovery** **/%**	**Purification Fold**
Crude Enzyme	67.4	662	0.102	100	1.00
Ammonium Sulfate Precipitation	45.0	162	0.277	66.8	2.72
DEAE Sepharose Fast Flow	16.8	27.0	0.623	24.9	6.11
Superdex-200	5.92	1.75	3.37	8.78	33.0

## Data Availability

The original contributions presented in this study are included in the article. Further inquiries can be directed to the corresponding author.
